# Small molecule targeting NaV1.7 via inhibition of the CRMP2-Ubc9 interaction reduces pain in chronic constriction injury (CCI) rats

**DOI:** 10.1080/19336950.2021.2023383

**Published:** 2022-01-05

**Authors:** Jiahe Li, Harrison J. Stratton, Sabina A. Lorca, Peter M. Grace, Rajesh Khanna

**Affiliations:** a Laboratories of Neuroimmunology, Department of Symptom Research, The University of Texas, Houston, Texas, USA; b Department of Pharmacology, College of Medicine, The University of Arizona, Tucson, Arizona, USA; c Comprehensive Pain and Addiction Center, The University of Arizona, Tucson, Arizona, USA

**Keywords:** Chronic pain, NaV1.7, CRMP2, SUMOylation, nociceptor, Ubc9

## Abstract

The voltage-gated sodium channel isoform NaV1.7 is a critical player in the transmission of nociceptive information. This channel has been heavily implicated in human genetic pain disorders and is a validated pain target. However, targeting this channel directly has failed, and an indirect approach – disruption of interactions with accessory protein partners – has emerged as a viable alternative strategy. We recently reported that a small-molecule inhibitor of CRMP2 SUMOylation, compound **194**, selectively reduces NaV1.7 currents in DRG neurons across species from mouse to human. This compound also reversed mechanical allodynia in a spared nerve injury and chemotherapy-induced model of neuropathic pain. Here, we show that oral administration of **194** reverses mechanical allodynia in a chronic constriction injury (CCI) model of neuropathic pain. Furthermore, we show that orally administered **194** reverses the increased latency to cross an aversive barrier in a mechanical conflict-avoidance task following CCI. These two findings, in the context of our previous report, support the conclusion that **194** is a robust inhibitor of NaV1.7 function with the ultimate effect of profoundly ameliorating mechanical allodynia associated with nerve injury. The fact that this was observed using both traditional, evoked measures of pain behavior as well as the more recently developed operator-independent mechanical conflict-avoidance assay increases confidence in the efficacy of **194**-induced anti-nociception.

The sensation of pain begins in the periphery where free nerve endings in the skin, equipped with a battery of specialized receptors, detect the presence of noxious stimuli [1,2]. Upon activation, these receptors cause small membrane depolarizations known as generator potentials [3]. When generator potentials are of sufficient magnitude, an action potential is triggered that relays this pain relevant information along the neuronal projections of primary afferent neurons that reside in the dorsal root ganglia (DRG) to the central terminals in the spinal cord dorsal horn [4]. The voltage-gated sodium channel NaV1.7 is expressed in the nociceptive afferent fibers in the skin as well as in the presynaptic terminals of the spinal cord dorsal horn and the cell bodies of DRG neurons [5]. Due to its unique biophysical properties – hyperpolarized activation voltage and slow entry into closed-state inactivation – NaV1.7 sets the threshold for action potential generation [6,7]. A crucial role for NaV1.7 in pain detection and transmission is supported by its involvement in multiple human pain conditions [8]. Patients with gain of function mutations in the gene encoding NaV1.7, *SCN9A*, present clinically with symptoms including excruciating episodic pain [9,10]. Conversely, patients with loss-of-function mutations to *SCN9A* produce a mutant NaV1.7 that results in the clinical manifestation of congenital insensitivity to pain [11]. These mutations in NaV1.7 that produce pain syndromes on opposite ends of the clinical spectrum have led to the widely accepted conclusion that NaV1.7 is a genetically validated pain target in humans.

This prime position in the nociceptive neurotransmission pathway makes NaV1.7 an appealing target for novel analgesic drug development. Targeting the voltage sensing segments of domain IV has led to compounds with excellent in vitro pharmacological properties, with some displaying 1000-fold selectivity for NaV1.7 over other voltage-gated sodium channel isoforms [12,13]. However, despite excellent in vitro pharmacology, attempts to directly inhibit NaV1.7 have failed to translate to the clinic [14]. This failure may be attributed to several disclosed factors, including failure to engage spinal NaV1.7, lack of recruitment of endogenous opioids, presumptive high target occupancy for analgesia, and flaws in the design of clinical trials testing these interventions [15,16]. Our group has pioneered an alternative approach that is focused on indirect targeting of NaV1.7 membrane trafficking through interference with NaV1.7 associated regulatory proteins [17–27].

One such regulatory protein, the cytosolic phosphoprotein collapsin response mediator protein 2 (CRMP2), has been intimately associated with the trafficking of receptors [28–32] and ion channels, including the presynaptic N-type voltage-gated calcium channel (CaV2.2) [23,33–48] and NaV1.7 [17–20,26,49–51]. We previously reported that knockdown of CRMP2 expression leads to a reduction in TTX-S current density, which includes NaV1.7, in cultured rat DRG sensory neurons [18]. Knockdown of CRMP2 expression did not affect TTX-R current density, which includes the NaV1.8 and NaV1.9 [18]. This selectivity in CRMP2-dependent voltage-gated sodium channel regulation suggested a control point somewhere in the signaling axis between CRMP2 and NaV1.7. The function of CRMP2 is controlled by its post-translational modification state [28,52–55]. We found that CRMP2-dependent regulation of NaV1.7 function is controlled by the addition of a small ubiquitin-like modifier (SUMO) to CRMP2 at Lys374 by the E2-SUMO conjugating enzyme Ubc9 [17,19]. Expression of a SUMO-null CRMP2, with Lys 374 mutated to an alanine, demonstrated a robust reduction in NaV1.7 currents measured in DRG sensory neurons. Other voltage-gated sodium channels were unaffected by co-expression of the SUMO-null CRMP2 [18]. Finally, we developed a CRMP2^K374A/K374A^ knock-in transgenic mouse incapable of CRMP2 SUMOylation [25]. Characterization of the phenotype developed by this mouse revealed reduced DRG voltage-gated sodium current density, reduced NaV1.7 membrane trafficking, and a profound resilience to the development of neuropathic pain [25]. Collectively, these results led to the conclusion that NaV1.7 function was under selective control by CRMP2 SUMOylation, which could therefore be targeted to induce analgesia.

This intriguing finding prompted us to develop a small molecule to prevent CRMP2 SUMOylation by interfering with the interaction with the SUMO conjugating enzyme Ubc9 [27]. A series of compounds were screened for in vitro inhibition of SUMO conjugation, which led to the identification of a benzoylated 2-(4-piperidinyl)-1,3-benzimidazole analog – compound **194** (Figure 1(a)) [27]. We reported that **194** specifically inhibits NaV1.7 currents in small DRG sensory neurons by interfering with channel trafficking and reducing NaV1.7 membrane localization [27]. Furthermore, this effect was conserved across species (mouse, rats, pigs, and humans) and the action of **194** was selective for NaV1.7 over other voltage-gated sodium channel isoforms. Importantly, **194** exhibited no reduction of sodium currents in DRG neurons from SUMO-null CRMP2^K374A/K374A^ transgenic knock-in mouse. Treatment with **194** also reduced presynaptic NaV1.7 in the spinal cord and reduced the frequency of excitatory post synaptic currents in the superficial layers of the spinal cord dorsal horn. Assessment of the *in vivo* effects of **194** demonstrated robust reversal of mechanical allodynia following spared nerve injury (SNI) and chemotherapy (paclitaxel)-induced peripheral neuropathy. Furthermore, the efficacy of **194** was enhanced with co-administration of a subtherapeutic dose of either morphine or gabapentin. Additionally, **194** did not affect locomotor activity, motor coordination, anxiety-like behavior, depressive-like behavior, or inhibit olfactory sensation. Together, these findings indicated that compound **194** is a potent inhibitor of mechanical allodynia in neuropathic models of pain with an excellent safety profile [27].

**Figure 1.**
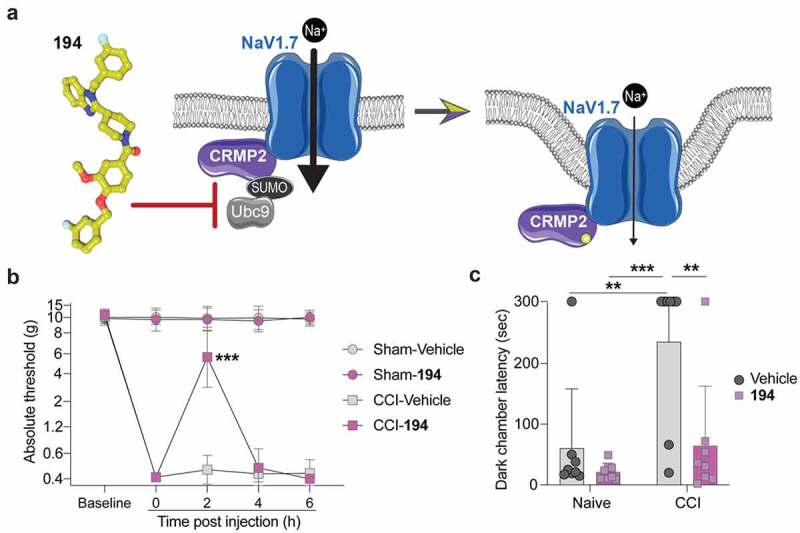
Compound **194** inhibits evoked and affective pain in rats with chronic constriction injury (CCI). (a) Schematic of the mode of action of compound **194** which uncouples the interaction between CRMP2 and the E2 SUMO-conjugating enzyme Ubc9 to prevent CRMP2 SUMOylation and reduces NaV1.7 cell-surface localization. This reduces sodium currents to alleviate pain. Image generated with BioRender. (b) Mechanical withdrawal thresholds were assessed in adult male rats before injury to establish a baseline and following injury to demonstrate the development of mechanical allodynia. Rats were then orally administered compound **194,** which reversed mechanical allodynia in the CCI group (pink squares) compared to the CCI animals given the vehicle (gray squares) (CCI-Vehicle vs. CCI-**194**, *p* = 0.0001 at 2 hours post injection). This effect peaked at 2 hours post administration before the animals returned to their postsurgery baseline sensitivity level. (c) Nociception was evaluated using the operator-independent mechanical conflict-avoidance assay. Naïve rats treated with **194** (pink squares) had the same latency to cross an aversive sharp surface as their vehicle treated counterparts (gray circles). Animals that had neuropathic pain induced by CCI had a profoundly increased latency to cross the aversive surface to escape the brightly lit enclosure (gray circles, right). Treatment with **194** significantly reduced the time to cross the aversive surface (pink squares, right) indicating reduced mechanical allodynia. (For B, multiple Mann–Whitney tests, *n* = 8 per group; for C, two-way ANOVA, naïve vehicle vs CCI vehicle, *p* = 0.0036; naïve **194** vs CCI vehicle, *p* = 0.0004; CCI vehicle vs CCI **194**, *p* = 0.0045, *n* = 8 per group).

However, the translational validity of evoked measures of nociception, such as assessment of mechanical hypersensitivity using von Frey filaments, has recently been questioned [56,57]. Furthermore, a recent report noted that establishing efficacy in a diverse array of pain models and operator-independent measures of nociceptive behaviors is essential for new analgesic candidates to successfully make the transition to the clinic [56]. Considering this report in favor of experimental diversity, we asked whether **194** could also be effective in reversing mechanical allodynia in other preclinical pain models, such as the chronic constriction injury (CCI) model of neuropathic pain [58], widely used peripheral nerve injury model of chronic neuropathic pain. Additionally, we employed the mechanical conflict-avoidance (MCA) assay, which is an operator-independent measure of nociception to validate our prior findings regarding the efficacy of **194** in preclinical pain models [59].

Oral administration of **194** significantly reversed mechanical allodynia observed in male rats following CCI surgery (Figure 1(b)). The allodynia was nearly completely reversed at 2 hours post administration of **194** and was followed by a return to the hypersensitive phenotype for the remainder of the test period (CCI-Vehicle vs. CCI-**194**, *p* = 0.0001 at 2 hours post injection). No effect was observed on mechanical withdrawal thresholds in the rats of the sham group treated with **194**. This indicates that **194** is only effective in animals that are in pain while sparing those that have no pain, which is consistent with the considerable safety profile observed in our previous study [27]. In addition to the traditional evaluation of nociception using von Frey filaments, we also assessed escape latency in the MCA assay. In this assay, rodents must escape a brightly lit chamber by passing through a corridor with nociceptive probes embedded in the floor. The probes are inconsequential to animals without pain but prove a major hurdle to those with hypersensitivity. The latency to escape the brightly lit chamber to the dark chamber on the other side of the aversive corridor is a measure of the pain sensitivity of the animal in response to the injury. Following CCI, animals demonstrated a significant delay in leaving the bright chamber and entering the dark chamber (Figure 1(c); naïve vehicle vs CCI vehicle, *p* = 0.0036; naïve **194** vs CCI vehicle, *p* = 0.0004; CCI vehicle vs CCI **194**, *p* = 0.0045), consistent with sensory dysfunction experienced due to CCI. Following oral treatment with **194**, the delay to enter the dark chamber was reversed (Figure 1(c), pink). This indicates that **194** induced anti-nociception in injured animals while producing no effect in uninjured animals. From this we can conclude that **194** ameliorated the neuropathic pain experienced in these rodents and allowed them to flee the brightly lit chamber unhindered.

These results support the findings reported by Cai et al. [27] that **194** can effectively reverse SNI- and chemotherapy-induced neuropathic pain and extends our understanding of **194**’s efficacy to another model of neuropathic pain, the chronic constriction injury of the sciatic nerve. The failure to translate preclinical findings to clinical therapeutics is thought to be in part based on overreliance on simple homogenous pain models [56]. Therefore, the finding that **194** reverses neuropathic pain in multiple different models of neuropathic pain is significant and supports the conclusion that **194** is capable of ameliorating persistent pain arising from different underlying pathophysiological mechanisms. Equally exciting is the reversal observed in the MCA assay, which, unlike evoked measures such as von Frey, assesses motivational and cognitive processing of pain-related information, and is less prone to operator bias. This increases the confidence in the finding that **194** can not only reverse neuropathic pain in traditional assays but is also capable of reversing complex pain behaviors using more modern and translatable assays, such as MCA. Furthermore, these results show that the effects of **194** are not limited to a single pain model or a single testing paradigm, which enhances overall confidence in the translational potential of this work. The results reported here are based on data from male rats and will likely need to be validated in females. However, we note that CRMP2 K374A knock-in mice, in which the SUMOylation site on CRMP2 at Lys 374 was replaced by an alanine, did not exhibit any sexual dimorphism in multiple parameters, including in long-term potentiation, compulsive- or depression-associated behaviors, response to thermal stimulation, excitatory neurotransmission in the spinal cord, licking responses to injection of the pan sodium-channel activator veratridine, and in their refractoriness to developing persistent mechanical allodynia fin the spared injury model of neuropathic pain [25].

The outcomes reported here are wholly congruent with our prior work regarding efficacy of inhibition of CRMP2 SUMOylation for the treatment of neuropathic pain and points toward a promising future for the development of **194** as novel therapy for treating neuropathic pain in humans [27,60].

## Methods

### Animals

Pathogen-free adult male Sprague Dawley rats (10 weeks old on arrival, Envigo, USA) were used. Rats were housed 2–3 per cage in a light- and temperature-controlled room (12:12-h light:dark cycle, lights on at 7:00 am) with food and water available *ad libitum*. No animals were excluded from this study for any reason. All procedures were approved by the MD Anderson Cancer Center Institutional Animal Care and Use Committee.

### Chronic constriction injury (CCI) surgery

Neuropathic pain was induced using the CCI model of sciatic nerve injury [58]. Surgery was performed at the mid-thigh level of the left hindleg as previously described [61,62]. In brief, animals were anesthetized with isoflurane. The shaved skin cleansed with povidone–iodine and 70% ethanol, and the surgery was performed aseptically. Four sterile chromic gut sutures (cuticular 4–0 WebGut, Patterson Veterinary, Devens, MA) were loosely tied around the gently isolated sciatic nerve. Animals were monitored postoperatively until fully ambulatory, prior to return to their home cage. Postoperative analgesia was not provided so as not to confound the endpoints under study. Naïve control rats were transported to the surgical suite at the same time that CCI surgeries were performed but were otherwise left undisturbed in their home cages.

### Drug administrations

Compound **194** was prepared in the appropriate diluent (10% DMSO, Sigma-Aldrich, St. Louis, MO; 10% tween-80, Sigma-Aldrich in saline, Hospira, Inc., Lake Forest, IL) and administered by oral gavage. **194** (2 mg/kg in 5 ml) or vehicle control was administered at 14 and 15 days postsurgery in a cross-over design. Dosing took place between 8:00 and 11:00 am. An independent experimenter performed dosing so as to blind the behavioral experimenter to the treatment groups.

### Mechanical allodynia

Rats received at least three 60-minute habituations to the test environment prior to behavioral testing. Rodents were placed in a small plexiglass enclosure on a mesh stand. The von Frey test [63] was performed as described [61,62]. Assessments were made before surgery, before dosing at 15 days post-surgery, and 2, 4, and 6 h post dosing. The behavioral responses were used to calculate absolute threshold (the 50% probability of response) by fitting a Gaussian integral psychometric function using a maximum-likelihood fitting method [64,65].

### Mechanical conflict-avoidance

Voluntary aversion to a noxious stimulus was assessed using a commercial 3-chambered apparatus, the Mechanical Conflict-avoidance System (Noldus, Leesburg, Virginia, USA). The apparatus presents rats with a choice in responding to two aversive stimuli – either to remain exposed to an aversive bright light in one chamber or to escape the light by crossing a middle chamber having a floor covered by a dense array of sharp probes, in order to reach a dark, safe chamber. Longer latencies to escape the light chamber indicate increased motivation to avoid the probes, and this escape latency is currently the most common measure of pain-related behavior in this test. We performed the operant mechanical conflict-avoidance test on rats as recently described [59,66]. Naïve rats were used in the control group, as we have previously shown that this assay is highly sensitive to postoperative pain after sham surgery [59]. The test was performed over 2 days, with each day consisting of three 300 s trials. Testing was performed between 8:00 am and 2:00 pm. In each trial 1) a rat was placed inside the light chamber with the lid closed, the light off, and the exit door closed; 2) after 20 seconds the light was turned on; 3) after 15 seconds the exit door was opened when (or if) the rat faced the exit; 4) the rat freely explored all 3 chambers in the apparatus for 300 s, 5) the rat was returned to its home cage, and 6) the device was thoroughly cleaned with 70% ethanol and distilled water in preparation for the next trial. On day 1 (third day of drug treatment), the probe height was set to 0 mm for all three trials. On day 2 (fourth day of drug treatment), the probe height was set to 0 mm for the first trial, and then raised to 4 mm for the second and third trials. The second and third trials were respectively performed 1.5 and 2 h after dosing. Data are presented as the latency to enter the dark compartment during the third trial on day 2.

### Statistical analyses

All data were first tested for a Gaussian distribution using a D’Agostino–Pearson test (Prism 9 Software, GraphPad, San Diego, CA). All data were analyzed using the non-parametric Kruskal–Wallis test followed by Dunn’s post-hoc test. Differences were considered significant if *p* ≤ 0.05.

Error bars in the graphs represent mean ± SD. All data were plotted in Prism 9.

## Data Availability

The authors confirm that the data supporting the findings of this study are available within the article.

## Statements and declarations

R. Khanna is the co-founder of Regulonix LLC, a company developing non-opioid drugs for chronic pain. In addition, R. Khanna 481 has patents US10287334 (Non-narcotic CRMP2 peptides targeting sodium channels for chronic 482 pain) and US10441586 (SUMOylation inhibitors and uses thereof) issued to Regulonix LLC. R. Khanna is also a co-founder of ElutheriaTx Inc., a company developing gene therapy approaches for chronic pain.
